# Mr. Watt, on Distortions of the Feet, &c.

**Published:** 1801-05

**Authors:** R. Watt

**Affiliations:** Paisley


### *To the Editors of the Medical and Phyſical Journal.*

GENTLEMEN,

IN the laſt Number of your valuable Journal, I ſee Mr. Sheldrake has again reſumed the diſpute, with regard to diſtorted limbs; and it is ſingular enough, that he ſhould now aſſign the *ſame* reaſon for doing this, that he did on a former occaſion for leaving it off*. In his laſt communication, however, he appears to have deſerted in a great meaſure the original diſpute, and to have ſubſtituted another in its place, from which he, no doubt, expects to acquire greater honour, or at any rate, to derive leſs diſgrace.

His original anſwer to my communication conſiſted of *three grand charges*, and a few common-place obſervations.—The firſt charge was, "That my invention was not original." In proof of this, three *reaſons* were adduced, all of which I have elſewhere refuted ſo fully, that Mr. S. has not ventured to ſay a *ſingle word in reply*. The ſecond charge was, "That the invention was uſeleſs." In proof of this, a number of *aſſertions* were adduced, all of which I have refuted fully by the beſt of all arguments, the teſt of experiment. And every day I ſee new proofs added to thoſe I have already brought forward. This part of the anſwer Mr. S. has alſo relinquiſhed as *indefenſible*; not a *ſingle word* is ſaid of it in his *laſt* communication. The third charge was, "That the hint thrown out in the end of my communication, with regard to the application of *a ſpring* to curvature in the bones of the leg, was *borrowed*, *copied*, or *purloined* from a work of *his*." This I alſo refuted as fully as I thought the importance of the ſubject required, or any reaſonable man could aſk; but as Mr. S. *ſeems ſtill unconvinced*, I beg leave to ſay a few words more on this ſubject.

I threw it out merely as a hint, which might be improved by others without ever having tried it myſelf; and in doing this I was not conſcious of doing injury or giving offence to any man;

---

* The reaſon Mr. S. aſſigned on a former occaſion for leaving off the diſpute, was my having uſed *perſonal abuſe*. Now, "It is to repel *ſuch reflections* ONLY that he has written this; and truſts that *you* (the Editors) will ſee the propriety of putting an end to *ſuch* diſputes, *which*, by the *conduct* of my *opponent*, have degenerated into *perſonal abuſe!*" When Mr. S. made this peroration, he ſeems to have foreſeen clearly, and dreaded the conſequences of his laſt communication!" He therefore, in order to get off with the victory, makes this ſingular requeſt to the Editors, that they publiſh *nothing more from his opponent!*

man; for I may fay, and I can fay it with truth, that I had never *feen* nor *heard of* Mr. Sheldrake's *fpecification*, nor his *Practical Effay*; nor did I know that fuch a man was *in exiftence* till I faw his anfwer to my communication. But as this *truth* can only be fubftantiated by *my affertion*, and as Mr. S. will probably require ftronger evidence, I may obferve that all the refemblance between the two contrivances is fimply this, *both ufe a fpring*. But of the particular method of ufing it, Mr. S. gives no account; he fays, it may be applied "by bandage or otherwife." In my communication, however, I give a *particular* account how it is fixed to the machine, how it is applied to the leg, and how it is to operate.

It is certainly a very *flender* proof of plagiarifm to fay that becaufe I ufe a *fpring*, and you ufe a *fpring*, therefore *you* muft have "borrowed, copied, or purloined" your idea from *me*. He might as well fay that becaufe a *watch* is put in motion by the action of a *spring*, a *firelock* is the *same*; therefore the man who invented the one muft have borrowed his ideas from the other; or as Mr. S. would elegantly exprefs it, "That he is a *mean* plagiary who *meanly* extracts from the works of others; thus meanly feeking to deprive them of what reputation may attach itfelf to their difcovery, and hoping to *purloin* that reputation to which *his conscience tells him he has no right!*" This is directly afferting that it is impoffible for two men to think in the fame way, without the one having borrowed his ideas from the other. This is contrary to Mr. S's *own* opinion; for he fays, that "the exiftence of fimilar paffages in different authors does not *neceffarily* imply plagiarifm in either."

If a bone, or other flexible body, be bent to the one fide more than is proper, would it not be very natural for a man to think or fay, that if preffure was made on the convex fide, and refiftance at the two ends, that the curve of courfe would be diminifhed. This is a principle known to every *mechanic*, and to every man who exercifes his reafon, and this is all Mr. S. claims as *his invention!* He fays this operation may be done by a fpring and bandage, or *otherwife*." In my communication, however, I ventured a little farther, and gave a drawing and defcription, not only of the machine and fpring by themfelves, but alfo of the manner in which they were to be applied to the leg.

But if Mr. S. fhould ftill doubt that I have borrowed my ideas from him, nothing certainly can be more convincing than his own words. "In the mean time," fays he, "I beg leave to obferve, that if a congrefs was affembled of all the *patients* who have been under my care, with their *friends*, and the *medical men* who have attended them, they would unanimoufly de-

 termine termine that Mr. W. has not *at present the most distant comprehension of my plan.*" And again, "Whoever will try (speaking prophetically) that particular modification of a spring which *he* recommends, will find *that it cannot answer the purpose he recommends it for.* I would ask Mr. S. how is it possible that I could have *borrowed*, *copied*, *purloined*, or by whatever term plagiarism may be designed," his ideas without having "the most *distant comprehension* of his plan?" Or how my plan, which he declared on a former occasion was the same as his, should be so *ineffectual*, while his is *efficacious?* These involve Mr. S. in a contradiction from which it will cost him *some trouble* to extricate himself.

Mr. Sheldrake surely does not claim the merit of having first invented a *spring*. No,—springs were known long before his day. It is only a *particular manner* of applying it to a *particular purpose*, that he claims as *his invention*; and of this *particular manner* he declares that I have not *at present the most distant comprehension*; therefore it was *impossible* I could *borrow*, *copy*, or *purloin* it from him. I may observe too, on the other ground *he* has laid down, that *my* invention cannot possibly be the same as *his*, or borrowed from *him*, since "it cannot answer the purpose it is recommended for," or as he elsewhere expresses it, "if any person will try the instrument described by Mr. Watt, he will find it *perfectly useless!*" If two instruments are the same, the effects they produce must also be the same. But if the effects are as *widely* different as Mr. S. has stated, it is surely no strained conclusion to infer *that the instruments are as widely different as the effects they produce*; and therefore the man who suggests the one can have no claim on the man who suggests the other. These are conclusions fairly drawn from Mr. S's *own premises*, and to which *he cannot possibly object.*

It would certainly be necessary when Mr. S. resumes the dispute, that he should make public through the medium of your Journal, *or otherwise*, a description and drawing of his plan, for it is evident he has not yet promulgated any thing on that head, or what he has promulgated is so obscure as not to be understood. For he declares I have not, "at present, the most distant comprehension of his plan." This, however, he may say is owing to the obtuseness of my intellect, by which I am not able to comprehend him; but he has said elsewhere,* "that it

* In a conversation that passed between Mr. S. and Mr. Pugh, concerning the cure of a distorted limb, Mr. S. says, that "Mr. P. was delighted to hear it muſt be by means ONLY KNOWN TO MYSELF, and by my *own* application." This puts it beyond all doubt, and ſhows the neceſſity of Mr. Sheldrake's bringing forward his invention; for how can a man diſpute on a ſubject of which he has not the moſt diſtant comprehenſion? Or how can Mr. S. blame others for purloining from him that which is known ONLY to himſelf. It muſt therefore depend entirely upon the *dictum* of Mr. S. whether my invention is the ſame as his or not, ſince HE *only knows what his invention is.*

Having diſcuſſed what belongs to the original diſpute concerning diſtorted limbs, I come now to conſider the charge of *perſonal abuſe*;† and as Mr. S. has complained ſo much of this particular, I ſhall trouble you with a few paſſages extracted from his laſt, which if they anſwer no better purpoſe, they will *at leaſt* convince your readers, and Mr. S. among the reſt, that no inconſiderable proportion of his communications is made up of *ſuch materials.* Among many other *epithets* and *inſinuations*, which it would be endleſs to enumerate, I am denominated by Mr. Sheldrake:

"*A Plagiary, a Purloiner, a Liar, a Coward, a Slanderer, a Dogmatiſt, an Impoſtor, a profeſſed Controvertialiſt, a Reproacher, a whimſical Perſon, and a Freebooter.*" ‡

When Mr. S. was writing an eſſay for the *expreſs purpoſe* of repelling ſuch *ſlanderous perſonal abuſe*, he ought to have couched it in language that was *at leaſt decent*, and not have proceeded to pluck the *mote* out of his neighbour's eye till he had firſt taken the BEAM out of his own. I cannot however help admiring hear that I thought a cure was not impracticable, and laboured ARDENTLY to perſuade *me* to inform *him* of the method I ſhould uſe, and put *him* in poſſeſſion of my inſtruments; but finding ME INFLEXIBLE, he, with *the affectation of much liberality, declared* he only ſought for the benefit of the patient, and therefore conſented that I ſhould proceed in my *own way.* Medical and Phyſical Journal, Vol. iv. p. 494. This proves in a very ſatisfactory manner that Mr. S. has not as yet made his plan public, nor, on account of his *inflexibility*, does he appear willing to do ſo.

† For the perſonal abuſe uſed againſt Mr. Sheldrake, ſee Medical and Phyſical Journal, Vol. v. p. 266, or rather Vol. iv. p. 498 to 503.

‡ Mr. S. ſays his patent extends only to England and Wales, and not to North Britain. This however is his own fault; for had his invention been of ſo much importance as he pretends, he could eaſily have extended it to Scotland alſo, and not have deprived any part of his country of ſo *invaluable a bleſſing!* But I may obſerve, that the *celebrity* of many inventions depends more on the *ſecrecy* with which they are preſerved from the *eye of the public* than on any *intrinſic value* in themſelves. Whether Mr. S's is of this deſcription or not, *no man* can ſay, ſince his invention is known *only to himſelf.*

admiring his ingenuity in being able to introduce ſo many of theſe *elegant figures of ſpeech* within ſuch narrow limits! In this particular he has certainly equalled, if not excelled, moſt authors who have written on the ſubject. I am, &c.

*Paiſley, March* 25, 1801. R. WATT.

---

